# Blocking the apoE/Aβ interaction ameliorates Aβ-related pathology in APOE ε2 and ε4 targeted replacement Alzheimer model mice

**DOI:** 10.1186/s40478-014-0075-0

**Published:** 2014-06-28

**Authors:** Joanna E Pankiewicz, Maitea Guridi, Jungsu Kim, Ayodeji A Asuni, Sandrine Sanchez, Patrick M Sullivan, David M Holtzman, Martin J Sadowski

**Affiliations:** Department of Neurology, New York University School of Medicine, New York, NY 10016 USA; Department of Psychiatry, New York University School of Medicine, New York, NY 10016 USA; Department of Biochemistry and Molecular Pharmacology, New York University School of Medicine, New York, NY 10016 USA; Department of Neurology, Washington University School of Medicine, St. Louis, MO 63110 USA; Knight Alzheimer’s Disease Research Center, Washington University School of Medicine, St. Louis, MO 63110 USA; Hope Center for Neurological Disorders, Washington University School of Medicine, St. Louis, MO 63110 USA; Department of Neuroscience, Mayo Clinic College of Medicine, Jacksonville, FL 32224 USA; Department of Medicine (Geriatrics), Duke University School of Medicine, Durham, NC 27710 USA; Durham VA Medical Center’s Geriatric Research, Education, and Clinical Center, Durham, NC 27710 USA; New York University School of Medicine, Alexandria East River Science Park, 450E 29th St., Room 830, New York, NY 10016 USA

**Keywords:** Apolipoprotein E, Alzheimer’s disease, β-amyloid, Neurodegeneration, Therapy

## Abstract

Accumulation of β-amyloid (Aβ) in the brain is essential to Alzheimer’s disease (AD) pathogenesis. Carriers of the apolipoprotein E (APOE) ε4 allele demonstrate greatly increased AD risk and enhanced brain Aβ deposition. In contrast, APOE ε2 allele carries show reduced AD risk, later age of disease onset, and lesser Aβ accumulation. However, it remains elusive whether the apoE2 isoform exerts truly protective effect against Aβ pathology or apoE2 plays deleterious role albeit less pronounced than the apoE4 isoform. Here, we characterized APP_SW_/PS1_dE9_/APOE ε2-TR (APP/E2) and APP_SW_/PS1_dE9_/APOE ε4-TR (APP/E4) mice, with targeted replacement (TR) of the murine Apoe for human ε2 or ε4 alleles, and used these models to investigate effects of pharmacological inhibition of the apoE/Aβ interaction on Aβ deposition and neuritic degeneration. APP/E2 and APP/E4 mice replicate differential effect of human apoE isoforms on Aβ pathology with APP/E4 mice showing a several-fold greater load of Aβ plaques, insoluble brain Aβ levels, Aβ oligomers, and density of neuritic plaques than APP/E2 mice. Furthermore, APP/E4 mice, but not APP/E2 mice, exhibit memory impairment on object recognition and radial arm maze tests. Between the age of 6 and 10 months APP/E2 and APP/E4 mice received treatment with Aβ12-28P, a non-toxic, synthetic peptide homologous to the apoE binding motif within the Aβ sequence, which competitively blocks the apoE/Aβ interaction. In both lines, the treatment significantly reduced brain Aβ accumulation, co-accumulation of apoE within Aβ plaques, and neuritic degeneration, and prevented memory deficit in APP/E4 mice. These results indicate that both apoE2 and apoE4 isoforms contribute to Aβ deposition and future therapies targeting the apoE/Aβ interaction could produce favorable outcome in APOE ε2 and ε4 allele carriers.

**Electronic supplementary material:**

The online version of this article (doi:10.1186/s40478-014-0075-0) contains supplementary material, which is available to authorized users.

## Introduction

Alzheimer’s disease (AD) is a familial and sporadic neurodegenerative disease. Its neuropathological hallmarks include parenchymal plaques and vascular deposits of β-amyloid (Aβ), intraneuronal paired helical filaments composed of hyperphosphorylated tau and ubiquitin, widespread loss of synapses and neurons, and the appearance of reactive astrocytes and microglia. Converging lines of evidence derived from genetic, neuropathological, biomarker, and transgenic animal studies implicate disturbance of Aβ homeostasis leading to its progressive accumulation in the brain as a driving mechanism of AD pathogenesis (reviewed in [1,2]). Aβ is a 39- to 43-residue-long hydrophobic peptide, which readily self-aggregates into synaptotoxic oligomers and thioflavin-S (Th-S)-binding fibrils. Mutations in amyloid precursor protein (APP) or presenilin (PS) 1 and 2 genes found in familial AD cases increase total Aβ secretion or alter a ratio of Aβ_1-40_:Aβ_1-42_ production, resulting in increased secretion of the more toxic and aggregation-prone Aβ_1-42_ [1]. In the far more prevalent sporadic AD, the mechanism(s) underlying disturbance of Aβ homeostasis are less obvious, but inheritance of the apolipoprotein (apo) E isoforms has been thus far established by numerous independent studies as the strongest genetic factor modulating overall risk of occurrence of the disease and age of onset (reviewed in [3]). ApoE is a 34-kDa lipid carrier protein, which in the brain is produced by astrocytes and secreted as nascent, high-density lipoprotein-like particles [4]. Three major isoforms of apoE are encountered in humans, differing by the occurrence of cysteine and arginine in positions 112 and 158: apoE2 (Cys112, Cys158), apoE3 (Cys112, Arg158), and apoE4 (Arg112, Arg158). These single-amino-acid variations in the apoE sequence have serious effects on its properties, with apoE2 having reduced binding affinity for the low density lipoprotein (LDL) receptor [5] and apoE4 exhibiting an intrinsic domain interaction that alters its lipid-binding properties and results in an increased risk of hyperlipidemia and atherosclerosis [4]. Inheritance of the APOE ε4 allele is the strongest known risk factor for sporadic AD and shows directs association with increased Aβ plaque load and reduced age of disease onset [6-8]. Conversely, the APOE ε2 allele appears to delay, and reduce the relative risk of developing AD and is associated with a lower burden of Aβ deposits compared with the most common APOE ε3 allele [9,10]. To provide a mechanistic explanation for the variable effect of apoE isoforms on Aβ accumulation and overall disease risk, it has been proposed that they produce a differential effect on Aβ clearance and Aβ aggregation and deposition [3]. However, it remains unclear whether the effect of apoE2 on Aβ pathology is truly protective, or whether apoE2 merely plays deleterious role albeit less pronounced than that of apoE4. To exert its promoting effect on Aβ aggregation and brain deposition, apoE directly interacts with Aβ, binding to its amino-acid residues 12–28 [11,12]. In this study, we analyzed long-term outcomes of the pharmacological disruption of the apoE/Aβ interaction in the background of apoE2 or apoE4, which show differential effect on Aβ pathology and AD morbidity. For this purpose we used APP_SW_/PS1_dE9_ AD transgenic (Tg) model mice with targeted replacement (TR) of both murine Apoe alleles with the human APOE ε2 or APOE ε4 alleles, which continued to be expressed under the control of the endogenous mouse Apoe promoter [13]. As previously shown, human APOE alleles targeted to APP_SW_/PS1_dE9_ mice reproduce their differential effect on the magnitude of Aβ plaque load [13] but do not delay onset of Aβ deposition compared to APP_SW_/PS1_dE9_ mice expressing native murine apoE [14]. Since these APP_SW_/PS1_dE9_/APOE ε2-TR and APP_SW_/PS1_dE9_/APOE ε4-TR lines (hereafter designated as APP/E2 and APP/E4; respectively) are novel AD Tg animal models, their behavioral and biochemical characterization was undertaken as a part of this study. To pharmacologically disrupt the apoE/Aβ interaction, we used a previously characterized non-toxic, synthetic peptide Aβ12-28P, which is homologous to the apoE binding motif within the Aβ sequence [12,13,15]. In Aβ12-28P, proline replaces valine in the 18 position and the peptide was synthesized with D-amino acids and end-protected for extended serum half-life and increased BBB permeability [12]. Aβ12-28P inhibits apoE4/Aβ binding with K_I_ = 12.9 nM and neutralizes the promoting effect of apoE on Aβ fibrillization *in vitro*, but has no effect on self-assembly of Aβ in the absence of apoE [15]. Therefore, this study also evaluated the prospects for disrupting the apoE/Aβ interaction in human population diversified by the occurrence of various APOE alleles.

## Material and methods

### Materials and reagents

Unless stated otherwise all reagents and antibodies were purchased from Sigma-Aldrich (St. Louis, MO). Aβ12-28P, Aβ_1-40_, and Aβ_1-42_ peptides were custom synthesized and purified in the WM Keck Proteomic Facility of Yale University (New Haven, CT) in the laboratory of Dr. James I. Elliott as previously described [12,13].

### Animals

All mouse care and experimental procedures were approved by Institutional Animal Care and Use Committees of the New York University School of Medicine and the Washington University School of Medicine. APP/E2 and APP/E4 mice were generated by crossing-breeding of APP_SW_/PS1_dE9_ mice [14] with APOE ε2-TR or APOE ε4-TR mice [16,17] as previously described [13]. APOE ε2-TR or APOE ε4-TR mice retain regulatory sequences of the mouse Apoe gene and the noncoding murine exon 1 surrounding the inserted human exons 2′, 3′, and 4′ [17,18] and therefore, they express the human apoE protein at physiological levels. Both lines were maintained by mating APP/E2 and APP/E4 founders with APOE ε2-TR and APOE ε4-TR animals, respectively. Husbandry and genotyping was performed as previously described [13]. Mice were maintained in a barrier facility with a 12/12-h light/dark cycle and ad libitum food and water access. Female APP/E2 and APP/E4 mice received treatment with vehicle or Aβ12-28P from the age of 6 to 10 months. Aβ12-28P was diluted to 2 mg/mL in sterile phosphate buffered saline at pH 7.4 directly before injections and intraperitoneally administered three times a week (0.8 mg per injection). Vehicle treated mice received injections with phosphate buffered saline only. Immediately prior to concluding the study the animals were subjected to behavioral testing conducted following our published protocols [15,19,20]. Age and sex-matched littermates of APP/E2 and APP/E4 mice in which genotyping was negative for the APP_SW_/PS1_dE9_ transgene (hereafter designated as WT/E2 and WT/E4, respectively) were used as an apoE isoform background control during the behavioral and serological testing. After concluding the experiment, all animals were killed with an overdose of sodium pentobarbital (150 mg/kg). The blood was collected through a cardiac puncture and the serum was separated by centrifugation. Animals were transcardially perfused and their brains and internal organs collected for histopathological analysis as previously described [15].

### Histological processing, immunohistochemistry, and unbiased morphometric analysis

Series of 40 μm-thick serial brain sections evenly spaced every 400 μm along the entire rostro-caudal brain axis were stained with Th-S to visualize fibrillar Aβ deposits, with Gallyas silver stain to identify plaque-associated neuritic dystrophy [21], or immunohistochemically with monoclonal antibody (mAb) HJ3.4 raised against the N-terminus of Aβ (1:250) [13] or with mAb 3D12 (1:200) raised against apoE (Novus Biologicals; Littleton, CO). The intensity of HJ3.4 and 3D12 immunostaining was enhanced by 10 min pretreatment with 44% formic acid (FA) and the immunohistochemistry protocol was concluded using 3,3′-diaminobenzidine and Cy3, respectively. The load of Th-S and 3D12 immunopositive amyloid plaques in the neocortex and hippocampus was determined using unbiased sampling and automated image threshold analysis, as previously described [13,15]. The density of neuritic plaques was calculated by counting their total number on all serial sections from a Gallyas stained series and then dividing the number obtained by the cumulative area of all the neocortical or hippocampal cross-sectional profiles.

### Aβ ELISA

Cortical mantle and hippocampi were dissected out, homogenized, and subjected to diethylamine (DEA) and FA extractions as previously described [15]. Concentrations of Aβ peptides in DEA and FA brain extracts were determined by sandwich ELISAs using mAbs HJ2 and HJ7.4, which selectively discriminate between C-terminal configuration of Aβ_x−40_ and Aβ_x−42_, respectively; as coating antibodies (1 μg/well) [13] and biotinylated mAb 4G8 (1:2,000) (Covance; Princeton, NJ) directed against Aβ residues 17–24 as the detection antibody [22,23]. Since the N-terminal configuration of Aβ peptides measured by ELISA remained uncharacterized we referred to them as Aβ_x-40_ and Aβ_x-42_. ELISA readouts were converted to actual peptide concentrations based on readouts from standard curves prepared from FA-treated synthetic Aβ_1-40_ and Aβ_1-42_ peptides and multiplied by all dilutions made during DEA and FA extractions. Final values of brain concentrations of Aβ peptides were reported in reference to the total brain protein concentration, the latter determined in a neat brain homogenate using the bicinchoninic acid assay (Pierce Biotechnology Inc.; Rockford, IL). The concentration of Aβ oligomers was determined in the neat homogenate using a human aggregated β-amyloid ELISA kit (Invitrogen, Carlsbad, CA) [13].

### ApoE ELISA

Sandwich ELISA for apoE was performed using mAb 3D12 (1:2,000) as the capture antibody and biotinylated goat anti-human apoE polyclonal antibody (1:2,500) (Meridian Life Science, Inc.; Memphis, TN) as the detection antibody. ApoE concentrations were quantified in DEA and FA brain extracts. ELISA readouts were converted to apoE concentrations using standard curves prepared from recombinant human apoE2 or apoE4 (Leinco Technologies, Inc., St. Louis, MO) and multiplied by all dilutions made during DEA or FA extractions. The final brain apoE concentrations were reported in reference to the total brain protein concentration.

### Statistical analysis

RAM data were analyzed using one-way analysis of variance (ANOVA) followed by the Newman-Keuls post hoc test. All other data sets were compared using the unpaired Student’s *t* test with Welch’s correction following confirmation of normal data distribution by the Kolmogorov-Smirnov and Shapiro-Wilk tests. All statistical analyses were performed using GraphPad Prism v5.04 (GraphPad Software, Inc., La Jolla, CA).

## Results

### Treatment of APP/E2 and APP/E4 mice with Aβ12-28P

Female APP/E2 and APP/E4 mice received Aβ12-28P or vehicle through intraperitoneal injections from the age of 6 to 10 months. The treatment commenced at the start of Aβ plaque formation in these Tg lines. Veterinary staff closely monitored the animals undergoing treatment for any signs of toxicity. Monitoring parameters included body weight, physical appearance, changes in coat appearance, occurrence of unprovoked behavior, and blunted or exaggerated responses to external stimuli. APP/E2 and APP/E4 mice receiving vehicle and Aβ12-28P showed no differences in respect to those parameters compared to their WT/E2 and WT/E4 age and sex-matched littermates, respectively. Measurements of the immune response against Aβ in post-treatment sera of APP/E2 and APP/E4 mice showed no evidence of increased titer of anti-Aβ antibodies in IgG and IgM classes associated with Aβ12-28P treatment (Additional file 1: Figure S1A and B). This observation indicates that treatment outcomes of Aβ12-28P administration cannot be accounted for by an anti-Aβ vaccination effect.

### Effect of Aβ12-28P treatment on serum cholesterol and apoE levels

We also monitored the effect of Aβ12-28P treatment on the serum cholesterol and apoE levels. Ten-month-old APP/E2 mice showed a 5.1-fold higher total serum cholesterol level and a 13.2-fold higher serum apoE level than APP/E4 mice (*p* < 0.001) (Additional file 1: Figure S2A). These findings are consistent with type III hyperlipoproteinemia, previously described in human APOE ε2-TR mice [17]. Compared to the vehicle control mice, Aβ12-28P treatment was associated with a reduction of the total serum cholesterol level by 15.7% in APP/E2 animals and by 33.3% in APP/E4 animals (*p* < 0.05) (Additional file 1: Figure S2A). In addition, the total serum apoE level showed a mild but insignificant reduction with Aβ12-28P treatment in both APP/E2 and APP/E4 mice (Additional file 1: Figure S2B). Consistently with our previously published observations [15] these results indicate that Aβ12-28P exerts an effect on serum cholesterol metabolism in addition to targeting the apoE/Aβ interaction in the brain.

### Behavioral studies

**Figure 1:**
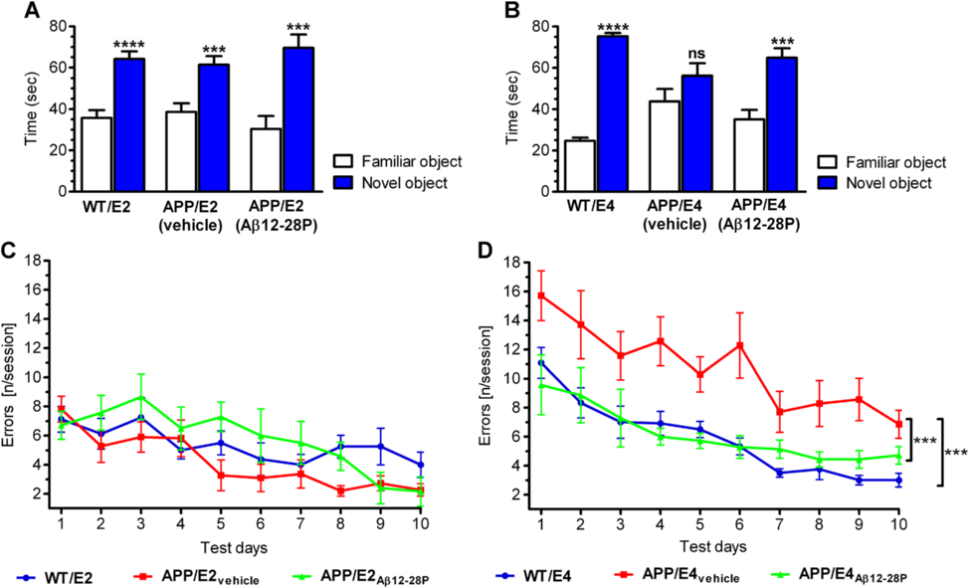
**Effects of Aβ12-28P treatment on the performance of APP/E2 and APP/E4 mice during the object recognition (ORT) and radial arm maze (RAM) tests.** Aβ12-28P treatment prevents memory deficit in APP/E4 mice. **(A and** **B)** Mean (±SEM) time spent interacting with familiar and novel objects during the retention session of the ORT (n = 8–12/group). ns: not significant, *** *p* < 0.01, **** *p* < 0.001, versus the familiar object (Student’s *t* test). **(C and** **D)** Mean (±SEM) number of errors per session plotted against testing days on the RAM (n = 7–12/group). **(C)**, ANOVA *p* = 0.139. **(D)**, ANOVA *p* = 0.0002, *** *p* < 0.001, versus APP/E4_vehicle_ (Newman-Keuls post-hoc test). Age and sex-matched wild-type (WT) littermates of APOE ε2 and ε4 background (WT/E2, WT/E4) were tested as controls in both tests.

The performance of vehicle- and Aβ12-28P-treated APP/E2 and APP/E4 mice was compared to those of sex and age matched WT/E2 and WT/E4 littermates, which received no treatment. Behavioral testing included the object recognition test (ORT), which tests animals’ long-term recognition memory, followed by the radial arm maze (RAM), which is a test of animals’ spatial working memory. Behavioral studies were conducted during the last 3 weeks of the treatment experiment and during the testing mice continued to receive vehicle or Aβ12-28P injections. During the acquisition session of the ORT, when the animals are allowed to explore freely the two identical objects presented in the testing arena, WT/E2, and vehicle- and Aβ12-28P-treated APP/E2 mice, equally interacted with both objects as expected of normal mice (data not shown). Following a 3-h interval, which the mice spent in their home cages, one of the two familiar objects was replaced with a novel one, and the behavior of the mice was then observed during the retention session. All three tested mice groups spent significantly more time exploring the novel object than the familiar one during the retention session (Figure 1A), consistent with normal rodent exploratory behavior. WT/E4 mice, and vehicle- and Aβ12-28P-treated APP/E4 mice, also interacted equally with two identical objects during the ORT acquisition session (data not shown). However, during the retention session, WT/E4 mice and Aβ12-28P-treated APP/E4 mice spent significantly more time exploring the novel object, while vehicle-treated APP/E4 mice failed to demonstrate significant preference toward the novel object (Figure 1B).

During RAM testing, WT/E2 mice, and vehicle- and Aβ12-28P-treated APP/E2 mice showed gradual improvement in performance on consecutive testing days, making comparable number of errors while navigating through the maze (Figure 1C). WT/E4 mice and vehicle- and Aβ12-28P-treated APP/E4 mice also showed gradual improvement in performance on consecutive days of RAM testing. However, vehicle-treated APP/E4 mice made significantly more errors than WT/E4 mice (*p* < 0.001) and Aβ12-28P-treated APP/E4 mice (*p* < 0.001), confirming the presence of memory impairment in APP/E4 mice at the age of 10 months (Figure 1D). Aβ12-28P-treated APP/E4 mice showed no significant difference from WT/E4 mice in the RAM testing, indicative of a therapeutic effect of apoE4/Aβ binding disruption *in vivo*.

### Analysis of Aβ plaque load

**Figure 2:**
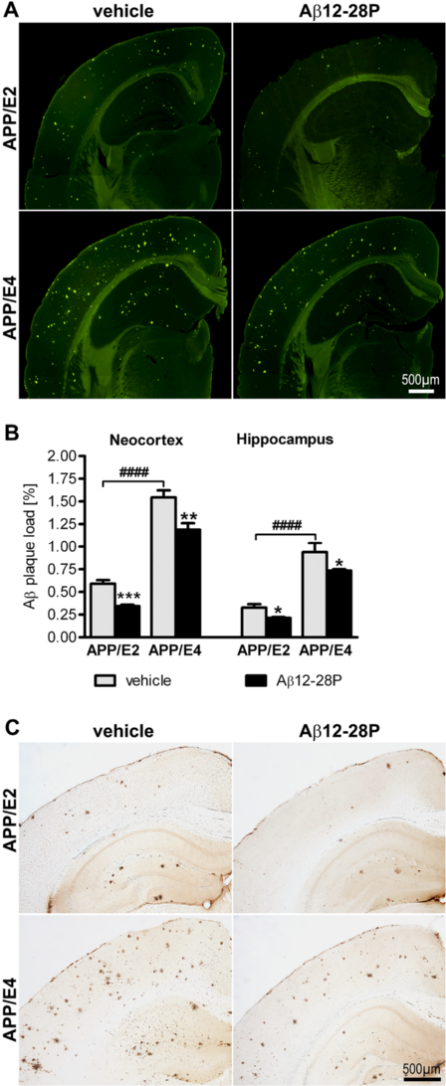
**Analysis of Aβ plaque load.** Aβ12-28P treatment reduces Aβ plaque formation in the neocortex and hippocampus of APP/E2 and APP/E4 mice. **(A)** Representative microphotographs of thioflavin-S-stained coronal brain sections from vehicle- and Aβ12-28P-treated APP/E2 and APP/E4 mice at the level of the dorsal hippocampus. **(B)** Mean (±SEM) thioflavin-S positive Aβ plaque load in the neocortex and the hippocampus (n = 7–8/group). * *p* < 0.05, ** *p* < 0.01, *** *p* < 0.001, vs. vehicle-treated mice of the same APOE background, (Student’s *t* test). ^####^ *p* < 0.0001, vehicle-treated APP/E2 mice vs. vehicle-treated APP/E4 mice (Student’s *t* test). **(C)** Representative microphotographs of coronal brain sections from vehicle- and Aβ12-28P-treated APP/E2 and APP/E4 mice showing the hippocampus, and somatosensory cortex, which were immunostained against the N-terminus of Aβ. Scale bars 500 μm **(A and** **C)**.

The load of Th-S positive Aβ plaques (percentage of cross-sectional area of an anatomical structure covered by the plaques) was quantified in the neocortex and the hippocampus on serial coronal brain sections. In vehicle-treated APP/E4 mice, the load of Th-S-stained plaques in the neocortex and the hippocampus was 2.6- and 2.9-fold higher than that in APP/E2 mice (*p* < 0.0001), (Figure 2A and B). Aβ12-28P treatment was associated with reduction of Aβ plaque load in the neocortex and the hippocampus in APP/E2 mice by 41.7% (*p* < 0.001) and 34.4% (*p* < 0.05), respectively, while in APP/E4 mice the reduction was 23.0% (*p* < 0.01) and 21.7% (*p* < 0.05), respectively. As Th-S reveals only fibrillar structure of Aβ plaques, we also stained serial brain sections with mAb HJ3.4 directed against the N-terminus of Aβ [13,24]. Differences in the load of Aβ plaque deposits shown by anti-Aβ immunohistochemistry among APP/E2 and APP/E4 mice treated with vehicle or Aβ12-28P corresponded to that revealed by Th-S staining (Figure 2C). A small number of Aβ deposits associated with brain and meningeal vessels was also seen in APP/E4 mice, however, their appearance was too sparse and variable from one brain section to the next to be reliably quantified.

### Biochemical analysis of brain Aβ levels

**Figure 3:**
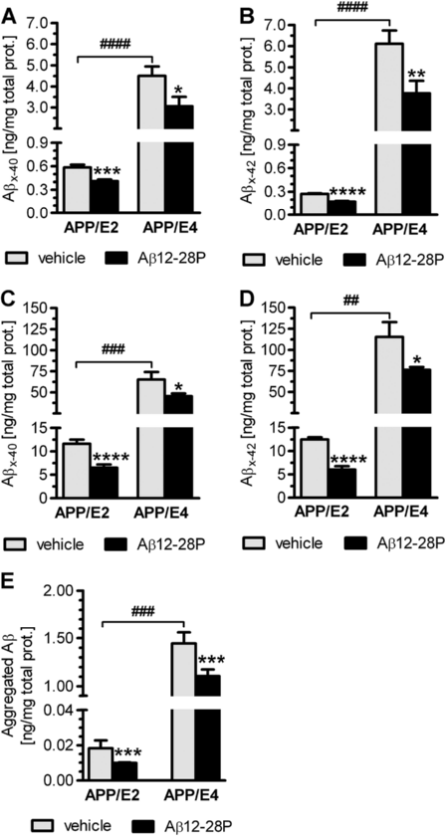
**Biochemical analysis of Aβ brain levels.** Aβ12-28P treatment reduces soluble and insoluble brain Aβ levels and those of aggregated Aβ in APP/E2 and APP/E4 mice. Levels of soluble (diethylamine-extractable) Aβ_x-40_ **(A)** and Aβ_x-42_ **(B)** and levels of insoluble (formic-acid-extractable) Aβ_x-40_ **(C)** and Aβ_x-42_ **(D)**. **(E)** Levels of aggregated Aβ species. All values are mean (±SEM) in 10–12 APP/E2 animals and 7–9 APP/E4 animals. **(A-E)**, * *p* < 0.05 ** *p* < 0.01, *** *p* <0.001, *** *p* <0.0001, versus the vehicle-treated mice of the same APOE background, (Student’s *t* test). ^##^ *p* < 0.01 ^###^ *p* < 0.001, ^####^ *p* < 0.0001, vehicle-treated APP/E2 mice vs. vehicle-treated APP/E4 mice (Student’s *t* test).

Brain Aβ_x-40_ and Aβ_x-42_ levels (please see The Methods section for explanation of Aβ peptide nomenclature used here) were measured following DEA and formic acid FA extractions, which release soluble Aβ and insoluble Aβ associated with Aβ plaques and vascular deposits, respectively. Striking differences between vehicle-treated APP/E2 and APP/E4 mice were noted. In APP/E4 mice, soluble Aβ_x-40_ and Aβ_x-42_ levels were 7.7-fold and 22.5-fold higher than those in APP/E2 mice (*p* < 0.0001), respectively; while insoluble Aβ_x-40_ and Aβ_x-42_ levels were 5.6-fold (*p* < 0.001) and 9.2-fold (*p* < 0.01) higher than those in APP/E2 mice, respectively (Figure 3A-D). Furthermore, the two lines significantly differed in the Aβ_x-40_:Aβ_x-42_ ratio. In APP/E2 mice the level of soluble Aβ_x-40_ was 2.1-fold higher than that of soluble Aβ_x-42_ (*p* < 0.0001), while in the APP/E4 mice the opposite relation was observed, with the level of soluble Aβ_x-42_ being 1.4-fold higher than that of soluble Aβ_x-40_ (*p* < 0.05). In APP/E2 mice, levels of insoluble Aβ_x-40_ and Aβ_x-42_ were similar, while in APP/E4 mice the level of insoluble Aβ_x-42_ was 1.8-fold higher than that of Aβ_x-40_ (*p* < 0.05). In both APP/E2 and APP/E4 mice, insoluble Aβ_x-40_ and Aβ_x-42_ levels were over 15-fold higher than those of soluble peptides (*p* < 0.001).

Aβ12-28P treatment was associated with a significant reduction in levels of soluble Aβ_x-40_ and Aβ_x-42_, which in APP/E2 mice were reduced by 30.1% (*p* < 0.001) and 35.8% (*p* < 0.0001), respectively; while in APP/E4 mice by 32.1% (*p* < 0.05) and 38.2% (*p* < 0.01), respectively (Figure 3A and B). Levels of insoluble Aβ_x-40_ and Aβ_x-42_ in Aβ12-28P-treated APP/E2 mice were reduced by 44.2% and 51.8%, respectively (*p* < 0.0001); while in Aβ12-28P-treated APP/E4 mice by 30.6% and 33.8%, respectively (*p* < 0.05) (Figure 3C and D). Furthermore, we measured the brains’ content of Aβ oligomers using ELISA, which utilizes the same mAb as both the capture and detection antibody, allowing four-member solid phase sandwiches to be formed only when aggregates containing multiple Aβ copies are present in the sample. As with other forms of Aβ, the concentration of Aβ oligomers in vehicle-treated APP/E4 mice was 79.2-fold higher than that in APP/E2 mice (*p* < 0.001). Aβ12-28P treatment reduced the level of Aβ oligomers by 46.1% and 23.5% in APP/E2 and APP/E4 mice, respectively (*p* < 0.001) (Figure 3E).

### Analysis of the brain apoE level and its accumulation within Aβ plaques

**Figure 4:**
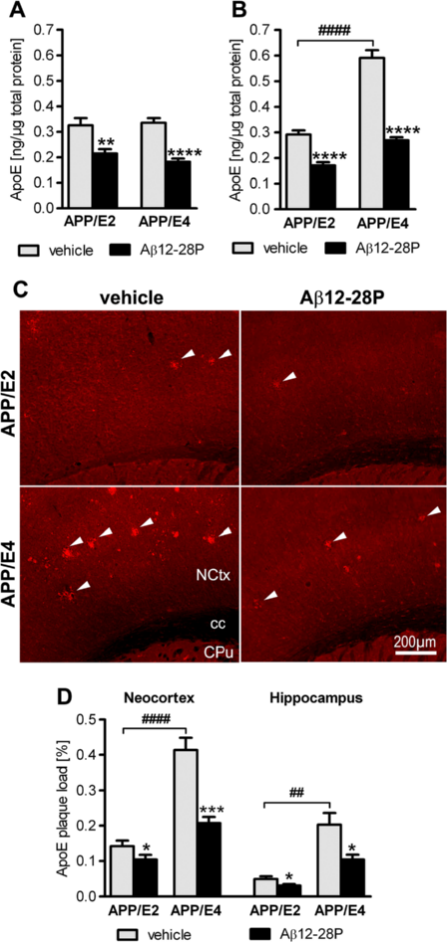
**Aβ12-28P treatment reduces soluble and insoluble brain apoE levels and apoE deposition in Aβ plaques in APP/E2 and APP/E4 mice.** Levels of soluble (diethylamine-extractable) human apoE **(A)** and those of insoluble (formic-acid-extractable) human apoE **(B)**. Values are mean (±SEM) in 11–12 animals of APP/E2 background and 8 animals of APP/E4 background. **(C)** Representative microphotographs of coronal sections through the sensorimotor cortex at the level of the posterior caudate-putamen from vehicle- and Aβ12-28P-treated APP/E2 and APP/E4 mice, which were immunostained against human apoE. White arrowheads indicate apoE positive deposits. **(D)** Mean (±SEM) human apoE positive plaque load in the neocortex and the hippocampus (n = 7–8/group). **(A, B, D)** * *p* < 0.05, ** *p* < 0.01, *** *p* < 0.001, *** *p* < 0.0001, versus vehicle-treated mice of the same APOE background (Student’s *t* test). ^##^ *p* < 0.01, ^####^ *p* < 0.0001, vehicle-treated APP/E2 mice vs. vehicle-treated APP/E4 mice (Student’s *t* test). Scale bar 200 μm **(C)**. Abbreviations: cc, corpus callosum; CPu, caudate-putamen; NCtx, neocortex.

Levels of soluble and insoluble human apoE were measured in brain homogenate extracted with DEA and FA, respectively. There was no significant difference in the level of soluble apoE between vehicle-treated APP/E2 and APP/E4 mice (Figure 4A). The insoluble apoE level was 2-fold higher in APP/E4 mice than in APP/E2 mice (*p* < 0.0001) (Figure 4B). Aβ12-28P treatment was associated with a 34.1% (*p* < 0.01) reduction in the soluble apoE level in APP/E2 mice and a 45.7% reduction in the level in APP/E4 mice (*p* < 0.0001). Insoluble apoE levels were reduced with Aβ12-28P treatment by 41.4% in APP/E2 mice (*p* < 0.0001) and by 54.6% in APP/E4 mice (*p* < 0.0001).

Furthermore, we quantified the load of apoE-positive plaque load, which also bound the amyloid dye Th-S. The load of apoE plaques was 2.9-fold higher in the neocortex (*p* < 0.0001) and 4.2-fold higher in the hippocampus (*p* < 0.01) in vehicle-treated APP/E4 mice than in the corresponding structures of vehicle-treated APP/E2 mice (Figure 4C and D). Aβ12-28P treatment was associated with a 26.9% reduction in the apoE-positive plaque load in the neocortex (*p* < 0.05) and a 37.5% reduction in the hippocampus (*p* < 0.05) in APP/E2 mice, and with a 49.9% (*p* < 0.001) and 48.5% (*p* < 0.05) reduction in the corresponding structures in APP/E4 mice, respectively.

### Neuritic plaques

**Figure 5:**
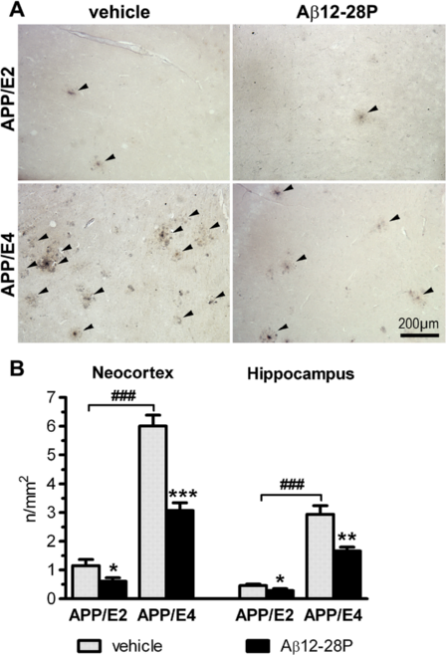
**Aβ12-28P treatment prevents neuritic degeneration in APP/E2 and APP/E4 mice. (A)** Representative microphotographs of coronal sections through the sensorimotor cortex at the level of the posterior caudate-putamen from vehicle- and Aβ12-28P-treated APP/E2 and APP/E4 mice stained with the Gallyas silver method. Black arrowheads indicate neuritic plaques revealed by the silver staining. **(B)** Mean (±SEM) of the numerical density of Gallyas-positive neuritic plaques in the neocortex and the hippocampus (n = 7–8/group). (B) * *p* < 0.05, ** *p* < 0.01, *** *p* < 0.001, versus vehicle-treated mice of the same APOE background (Student’s *t* test). ^###^ *p* < 0.001, vehicle-treated APP/E2 mice vs. vehicle-treated APP/E4 mice (Student’s *t* test). Scale bar 200 μm.

Neuritic dystrophy associated with Aβ plaques was revealed using the Gallyas silver stain (Additional file 1: Figure S3 A-E). In APP/E4 mice, the numerical density of neuritic plaques in the neocortex and the hippocampus was 5.2-fold and 6.5-fold higher than those in the corresponding structures of APP/E2 mice, respectively (*p* < 0.001) (Figure 5A and B). Aβ12-28P treatment reduced the neuritic plaques density in the neocortex and the hippocampus of APP/E2 mice by 47.1% and 38.5%, respectively (*p* < 0.05), while in APP/E4 mice by 48.8% (*p* < 0.001) and 43.6% (*p* < 0.01), respectively.

## Discussion

ApoE isoforms differentially modulate Aβ metabolism, Aβ accumulation, and AD morbidity in human subjects. We demonstrated here that APOE ε2 and APOE ε4 targeted replacement in APP_SW_/PS1_dE9_ AD Tg model mice reproduces the differential effect of encoded by these alleles apoE isoforms on various aspects of Aβ pathology and associated neurodegeneration. Ten-month-old APP/E2 mice showed no behavioral deficit compared to WT/E2 mice, despite a modest Aβ plaque load and Aβ oligomer level. In contrast, age and sex matched APP/E4 mice revealed a pronounced memory deficit along with a several-fold higher load of Aβ plaques and insoluble Aβ level, and nearly an 80-fold higher level of Aβ oligomers compared to APP/E2 mice. Oligomeric Aβ species have been shown to cause memory deficits in rodents [25] and are known for their synaptotoxic properties. This synaptotoxicity is associated with a reduction in the surface expression of various receptor proteins, including those forming the N-methyl-D-aspartate receptors [26,27], which are critical for generation of long-term potentiation in hippocampal synapses. We also found evidence for a differential effect of apoE2 and apoE4 isoforms on Aβ metabolism in APP_SW_/PS1_dE9_ mice. APP/E4 mice had significantly higher levels of soluble Aβ peptides than APP/E2 mice. In particular, the level of soluble Aβ_x-42_, which is considered more toxic and aggregation prone than Aβ_x-40_, was 22.5-fold higher in APP/E4 mice than in APP/E2 mice. Furthermore, in APP/E2 mice, the soluble Aβ_x-40_ level was 2.1-fold higher than that of Aβ_x-42_, while in APP/E4 the ratio was reversed, with the level of soluble Aβ_x-42_ being 1.4-fold higher than that of Aβ_x-40_. These striking differences in the brain levels of soluble Aβ_x-40_ and Aβ_x-42_ peptides, and the reversed ratio, between APP/E2 and APP/E4 mice are likely related to the well-established differential effect of apoE2 and apoE4 isoforms on Aβ brain clearance [28]. Increased soluble Aβ levels and a predominance of Aβ_x-42_ associated with the apoE4 isoform, produce a background promoting greater Aβ deposition and enhanced neurodegeneration. In fact, the load of Th-S positive Aβ plaques and the brain concentration of insoluble Aβ, which are associated with fibrillar Aβ deposits, were several-fold higher in APP/E4 mice than in APP/E2 mice. Enhanced Aβ deposition was, in turn, associated with a greater degree of neurodegeneration, as demonstrated by a several-fold higher density of neuritic plaques in APP/E4 animals as compared to APP/E2 animals. APOE ε4 targeted replacement was also associated with significantly greater co-deposition of apoE in Aβ plaques, as shown by the comparison of apoE-positive plaque load and the level of insoluble apoE between APP/E4 and APP/E2 mice. It has been previously demonstrated that co-deposition of apoE in Aβ plaques is a prerequisite for the occurrence of focal neuritic dystrophy [22,29].

Epidemiological evidence and evidence from neuropathological assessment of the impact of apoE isoforms on the brain Aβ load, have suggested that apoE2 exerts a protective effect against Aβ pathology and AD morbidity [3,9,10], while apoE4, in contrast, markedly enhances the disease process [7,8,30]. To directly examine whether apoE2 exerts a truly “therapeutic” effect or, on the contrary, is involved in promoting Aβ deposition, we systemically treated APP/E2 mice with Aβ12-28P, which disrupts the apoE/Aβ binding. Another objective of this study was to investigate whether an apoE/Aβ binding inhibitor would ameliorate Aβ pathology and associated neurodegeneration, as shown in an exaggerated form by the apoE4 isoform. We demonstrated that Aβ12-28P treatment produced significant reductions in the Aβ oligomer level, the Aβ plaque load, and the concentration of insoluble Aβ peptides, and ameliorated the neurodegenerative component of Aβ plaques in both APP/E2 and APP/E4 mice. These observations provide clear *in vivo* evidence that both apoE2 and apoE4 isoforms are involved in the process of Aβ aggregation and deposition and are associated with neurodegeneration, although the effect of apoE4 appears to be much stronger than that of apoE2. Our findings remain consistent with the effects of apoE knock-out noted in several AD Tg mouse models, where lack of apoE was associated with absence of fibrillar Aβ deposits and prevented neurodegeneration [13,29,31,32]. In addition to a marked reduction in Aβ deposition in Aβ12-28P-treated mice, we also noticed a significant reduction in the level of Aβ oligomers. It has been recently appreciated that apoE, especially its apoE4 isoform, facilitates the formation of Aβ oligomers [33,34]. Although it appears that enhanced Aβ oligomerization occurs in settings of elevated Aβ_x-42_ level, which is associated with the apoE4 background, there is experimental evidence to indicate that direct interaction between apoE and Aβ facilitates Aβ oligomeric assembly and that all three isoforms promote this process in the rank order E4 > E3 > E2 [33]. Reduced Aβ oligomer level following from disrupted apoE/Aβ binding appears to provide further evidence that the direct apoE/Aβ interaction enhances oligomeric assembly of Aβ and that treatment with an apoE/Aβ inhibitor is beneficial for preventing Aβ oligomerization and for ameliorating synaptotoxicty related to Aβ oligomers. Furthermore, we observed that along with reducing Aβ deposition, Aβ12-28P treatment also ameliorated apoE accumulation in the brain. It has been proposed that co-deposition of apoE with Aβ is an important factor promoting fibrillization of Aβ and plaque formation [22,23]. It has also been recognized that co-deposition of apoE in Aβ plaques promotes focal neurodegeneration in humans and in AD Tg mice [22,29,35]. Reduction of apoE deposition in Aβ12-28P-treated mice demonstrates an additional mechanism through which disruption of the apoE/Aβ interaction may ameliorate Aβ plaque formation and associated neurodegeneration. Similarly, it has been shown that Aβ accumulation in the brain of APP_SW_/PS1_dE9_ mice can be effectively reduced through targeting brain apoE with systemic anti-apoE passive immunization [36].

Clearance of soluble Aβ from the brain is critical in preventing its accumulation and there is evidence this process can be differentially regulated by apoE isoforms [28]. Several studies have shown that the bulk of soluble Aβ is cleared through its direct interaction with the LDL receptor family expressed by brain capillary endothelium [37], neurons [24,38], and astrocytes [39,40], therefore pharmacological disruption of the apoE/Aβ binding is unlikely to impair this process. What is more, since some of Aβ forms complexes with apoE [41], an apoE/Aβ antagonist can free up this apoE bound Aβ increasing soluble Aβ pool and promoting its clearance. It has been also proposed that apoE can indirectly modulate Aβ clearance through competing with Aβ for the same receptors [40]. This notion may explain enhanced Aβ clearance in the setting of apoE2, which is defective in LDL receptor binding [4,5]. Since we observed reduced levels of soluble apoE in Aβ12-28P-treated APP/E2 and APP/E4 mice, the treatment with an apoE/Aβ inhibitor may additionally facilitate Aβ clearance by reducing the levels of apoE competing with Aβ for the same receptor clearance pathway. In fact, in our study levels of soluble Aβ_x-40_ and Aβ_x-42_ peptides were significantly reduced in both Aβ12-28P-treated APP/E2 and APP/E4 mice providing evidence that the apoE/Aβ binding antagonist may enhance soluble Aβ clearance.

The accumulation of Aβ in the brain is considered to be a prime target of disease-modifying therapies for AD [2]. Although clinical trials of the Aβ-directed mAb Bapinezumab showed no clear cognitive benefits in patients with mild to moderate AD [42], the results also indicated that APOE ε4 carrier status may limit the response to anti-Aβ passive immunization [43,44], as well as being associated with an increased rate of microbleeds and vasogenic edema [45]. As the intention of upcoming clinical trials of Aβ-directed therapeutics is to target patients with early and prodromal AD [46,47], there is still a potential for differential responses to treatment among carriers of various APOE alleles. Our study provides evidence for efficacy of an agent disrupting the apoE/Aβ interaction in the setting of apoE2 and apoE4 isoforms. Therefore, such an agent could be applied in human populations diversified by apoE isoform status. Furthermore, as apoE/Aβ binding inhibitors would primarily target apoE promoting effect on Aβ assembly and deposition, they have the potential to be combined for additive efficacy with other Aβ-targeting strategies still under development, including β-secretase inhibitors and γ-secretase modulators inhibiting Aβ production, or with passive immunization, which promotes Aβ clearance from the brain and stimulates plaque clearance by microglia [48,49]. Results of this study justify efforts to develop clinically viable, small-molecule inhibitors of the apoE/Aβ interaction and test their therapeutic application, either as a monotherapy or in conjunction with other anti-Aβ approaches.

## Additional file

Additional file 1:
**Detailed results concerning detection of anti-Aβ antibodies, and quantification of cholesterol and apoE levels in the sera of vehicle and Aβ12-28P treated animals and detailed methods used for determination of thereof.** Demonstration of neuritic dystrophy associated with amyloid Aβ plaques.
