# Supplementary material for: Blocking the apoE/Aβ interaction ameliorates Aβ-related pathology in APOE ε2 and ε4 targeted replacement Alzheimer model mice
Source: Acta Neuropathol Commun. 2014 Jun 28;2:75. doi: 10.1186/s40478-014-0075-0 (PMC4174325; doi:10.1186/s40478-014-0075-0)
Supplement: Additional file 1: — Detailed results concerning detection of anti-Aβ antibodies, and quantification of cholesterol and apoE levels in the sera of vehicle and Aβ12-28P treated animals and detailed methods used for determination of thereof. Demonstration of neuritic dystrophy associated with amyloid Aβ plaques. [file 40478_2014_75_MOESM1_ESM.docx]

**Supplementary Materials and Methods**

Detection of anti-Aβ antibodies in the serum

Sera of vehicle- and Aβ12-28P-treated APP/E2 and APP/E4 mice were collected at the conclusion of the experiment, diluted 1:500, and added to ELISA plates coated with synthetic Aβ_1–40_ (50 ng/well). The plates were washed and incubated with specific anti-mouse IgG or IgM mAbs conjugated with horseradish peroxidase (Amersham Biosciences, Piscataway, NJ) at a 1:5,000 dilution, followed by the addition of 3,3,5,5-tetramethylbenzidine substrate. The color reaction was stopped with 2M sulfuric acid, and the absorbance was read at a wavelength of 450 nm. A mixture of HJ3.4 and HJ2 mAbs directed against the N- and C-termini of Aβ_1–40_, which were serially diluted from 1:100 to 1:3,125,000 from the initial stock of 0.5 mg/mL each, were used to develop a standard curve. Sera of age- and sex-matched WT/E2 and WT/E4 mice, which do not produce human Aβ, were used to determine nonspecific background adsorption. Sera immunoreactivities were compared by means of one-way analysis of variance (ANOVA) followed by the Tukey-Kramer post hoc test using GraphPad Prism v5.04 (GraphPad Software, Inc., La Jolla, CA).

Serum cholesterol concentration

The total serum cholesterol concentration was measured using a standard enzymatic assay based on the Cholesterol E kit (Wako Diagnostics, Richmond, VA).

Serum apoE concentration

Sandwich ELISA was used to determine the apoE concentration in the serum, using mAb 3D12 (1:2,000) as the capture antibody and biotinylated goat anti-human apoE polyclonal antibody (1:2,500) (Meridian Life Science, Inc., Memphis, TN) as the detection antibody. ELISA readouts were converted to the actual apoE concentrations based on standard curves prepared from recombinant human apoE2 or apoE4 (Leinco Technologies, Inc., St. Louis, MO) and multiplied by serum dilutions, which for APP/E2 and APP/E4 mice were 1:20,000 and 1:16,000, respectively.

**Supplementary data**


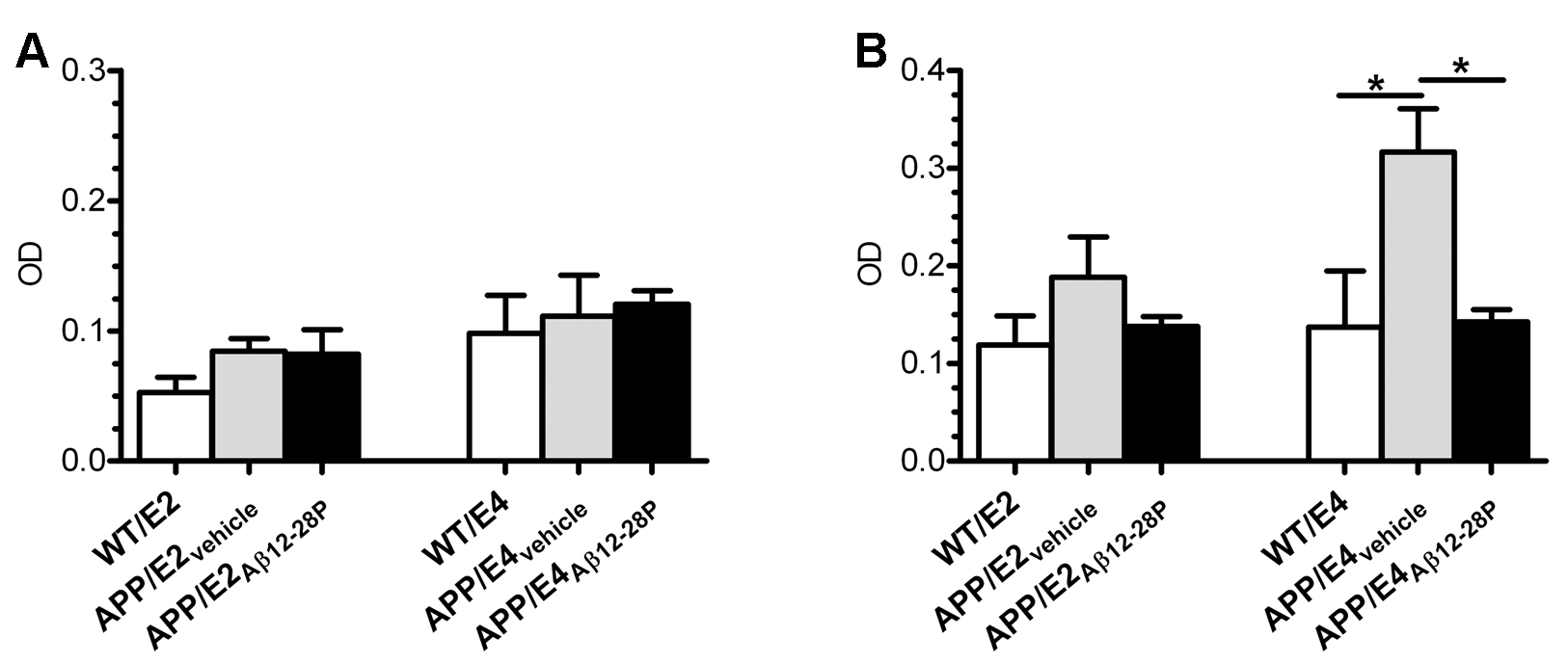


**Fig. S1** Aβ12-28P treatment is not associated with anti-Aβ immune response. Measurements of the anti-Aβ immune response in IgM (A) and IgG (B) antibody classes in post-treatment sera of vehicle- and Aβ12-28P-treated APP/E2 and APP/E4 mice. No evidence for immune response against Aβ in the IgM antibody class was found in APP/E2 or APP/E4 mice as compared to the background immunoreactivity of sera from age-, sex-, and apoE-background-matched wild-type (WT) mice, i.e., WT/E2 and WT/E4, respectively. Likewise, there was no evidence for anti-Aβ IgG class response in APP/E2 mice. Modest anti-Aβ immune response in the IgG class was detected among vehicle-treated APP/E4 mice as compared to WT/E4 mice, but not in those treated with Aβ12-28P. Mean concentration of anti-Aβ antibodies in the sera of vehicle-treated APP/E4 mice was estimated at 132 ± 27 ng/mL based on a standard curve prepared from serial dilution of HJ2 and HJ 3.4 mAbs. (A and B) Mean (±SEM) of optical density (OD) ELISA readouts from sera of untreated WT/E2 and WT/E4 and vehicle- or Aβ12-28P- treated APP/E2 and APP/E4 mice (n = 6–8). * *p* < 0.05 for the Tukey-Kramer Multiple Comparisons Test following one-way analysis of variance (ANOVA), which gave *P* = 0.011 for the IgG class response in ε4 background mice


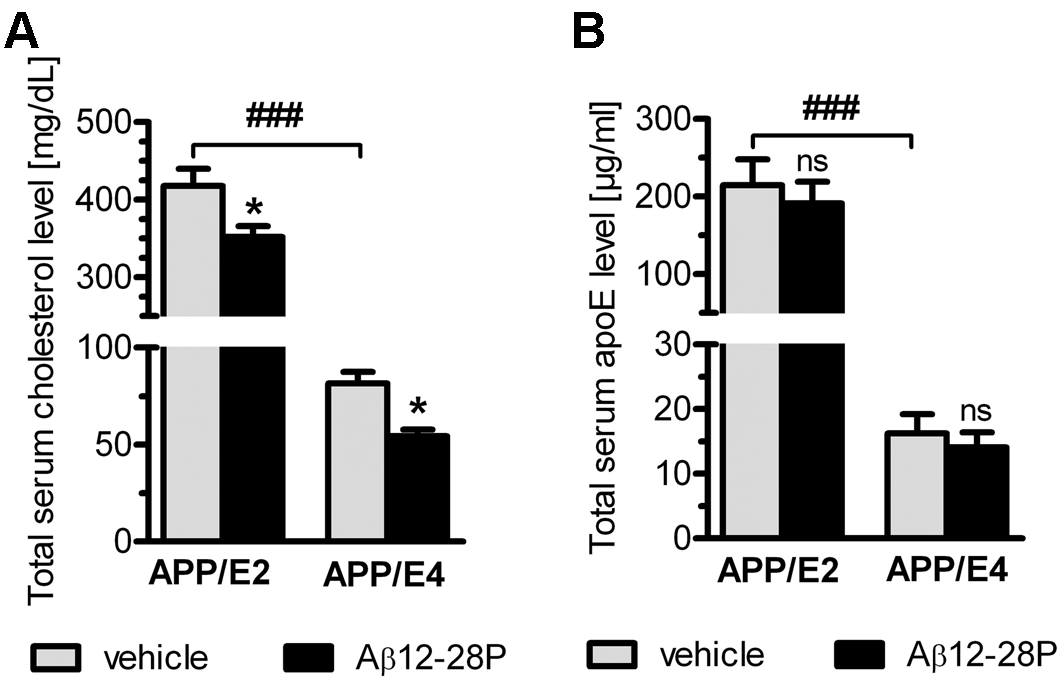


**Fig. S2** Effect of Aβ12-28P treatment on the total cholesterol and apoE serum levels. Aβ12-28P treatment is associated with significant reduction in the serum cholesterol level in APP/E2 and APP/E4 mice. Total serum cholesterol level (A) and total serum apoE levels (B) were measured in vehicle- and Aβ12-28P-treated APP/E2 and APP/E4 mice at the conclusion of the Aβ12-28P treatment experiment. All values are mean (±SEM) in 7–10 animals of APOE ε2 background and 6–7 animals of APOE ε4 background. (A and B) ns: not significant, * *p* < 0.05, vs. the vehicle-treated mice of the same APOE background (Student’s *t* test). ^###^ *p* < 0.001, vehicle-treated APP/E2 vs. vehicle-treated APP/E4 (Student’s *t* test)


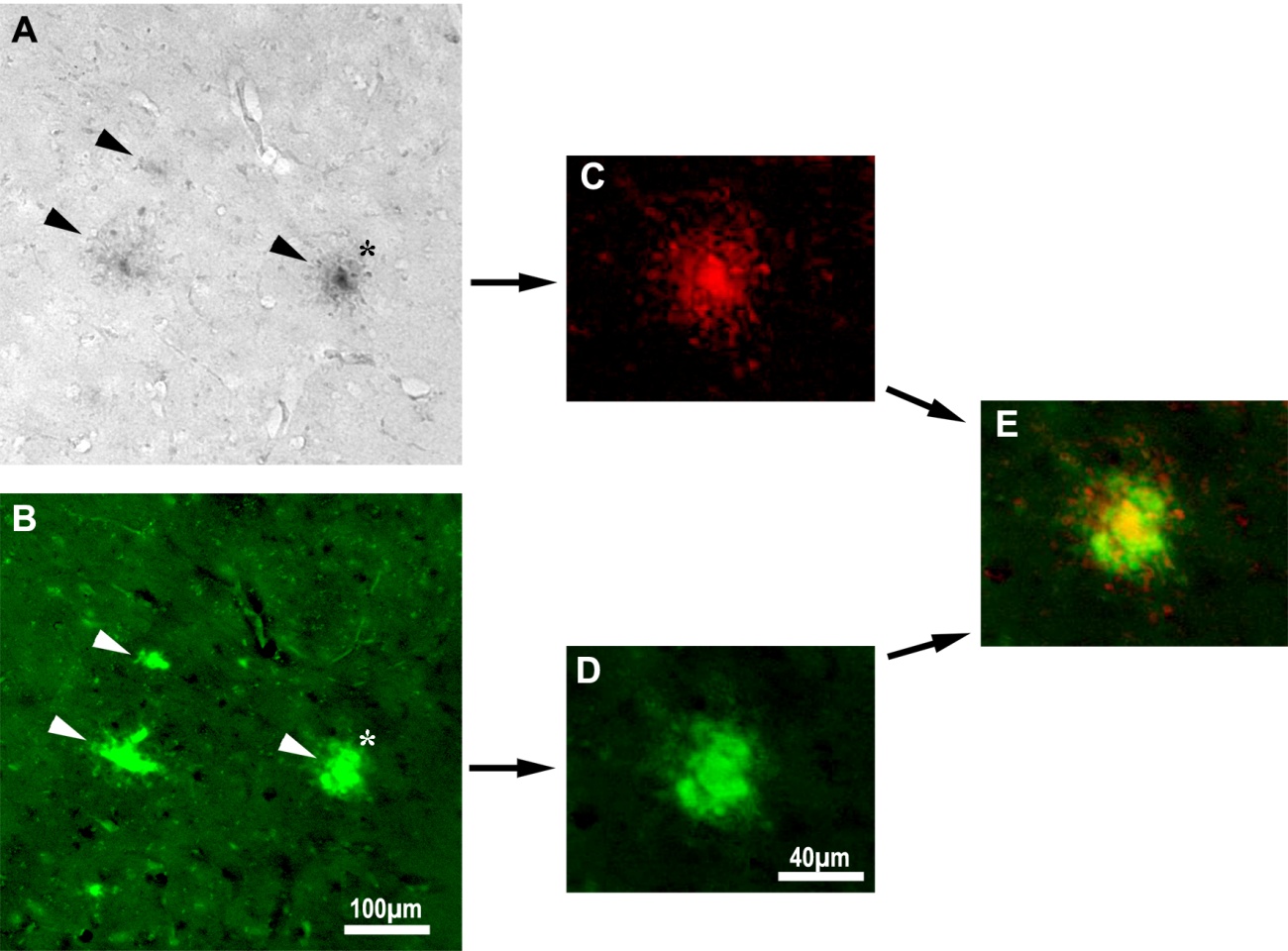


**Fig. S3** Demonstration of neuritic dystrophy associated with amyloid Aβ plaques. Representative microphotographs of the cerebral cortex in coronal section from a vehicle-treated APP/E4 mouse; the section was double-stained with Gallyas silver staining (A*)* and thioflavin-S (B) and photographed under bright-field and fluorescence microscopy, respectively. Arrowheads indicate matching neuritic and amyloid components of senile plaques, revealed by Gallyas and thioflavin-S staining, respectively. High-power images of a neuritic plaque digitally converted to red (C) and its corresponding amyloid component (D), which are indicated by asterisks in (*A*) and (*B*), respectively. (E) Merged image of (C) and (D) showing co-localization of dystrophic neurites (red) and amyloid (green). Scale bars 100 μm (A and B) and 40 μm (C–E)
